# Draft Genome Sequence and Gene Annotation of the Uropathogenic Bacterium *Proteus mirabilis* Pr2921

**DOI:** 10.1128/genomeA.00564-16

**Published:** 2016-06-23

**Authors:** F. M. Giorello, V. Romero, J. Farias, P. Scavone, A. Umpiérrez, P. Zunino, J. R. Sotelo Silveira

**Affiliations:** a Department of Genomics, Instituto de Investigaciones Biológicas Clemente Estable, Montevideo, Uruguay; b Sequencing Platform, Instituto de Investigaciones Biológicas Clemente Estable, Montevideo, Uruguay; c Department of Proteins and Nucleic Acids, Instituto de Investigaciones Biológicas Clemente Estable, Montevideo, Uruguay; d Department of Microbiology, Instituto de Investigaciones Biológicas Clemente Estable, Montevideo, Uruguay

## Abstract

Here, we report the genome sequence of *Proteus mirabilis* Pr2921, a uropathogenic bacterium that can cause severe complicated urinary tract infections. After gene annotation, we identified two additional copies of *ucaA*, one of the most studied fimbrial protein genes, and other fimbriae related-proteins that are not present in *P. mirabilis* HI4320.

## GENOME ANNOUNCEMENT

The bacterium *Proteus mirabilis* can cause severe complicated urinary tract infections (UTIs), especially in patients with indwelling catheters. *P. mirabilis* expresses several fimbrial proteins that are possibly related to its uropathogenic capacity (1, 2). In particular, the uroepithelial cell adhesin (UCA) fimbria seems to be important in the colonization of the urinary tract (2). In this work, we sequenced the genome of *P. mirabilis* Pr2921 (3) isolated from the urine from a patient with a UTI.

The DNA of *P. mirabilis* Pr2921 was extracted using standard protocols. Whole-genome sequencing was performed using the Ion Torrent PGM platform, yielding 406,610 unpaired reads. The genome assembly was carried out with SPAdes assembler version 3.1.0 (4) using the IonHammer read correction tool. After removing redundancies, we obtained 56 contigs (>500 bp), which were assembled into 49 nonredundant scaffolds (>500 bp), with a total length of 3,924,384 bp, a G+C content of 38.66%, and an *N*_50_ of 200,766 bp. More than 95% of the reads were used in the final assembly.

Through Rapid Annotations using Subsystems Technology (RAST) (5), 3,651 coding sequences (CDSs), 92 tRNAs, and 16 rRNAs were predicted. A total of 3,315 CDSs were successfully annotated using blastp (*E* value cutoff, 1e - 10) against *P. mirabilis* HI4320 (6). Of the 336 remaining genes, 236 were annotated using the NCBI nonredundant protein database as a reference. More than 85% of the CDS predictions presented at least one Pfam domain.

We identified the four most studied *P. mirabilis* fimbriae, mannose-resistant/*Proteus*-like (MR/P), *P. mirabilis* fimbriae (PMF), uroepithelial cell adhesin (UCA), and ambient-temperature fimbria (ATF)*.* The percentage of aminoacidic divergence for these four proteins between our strain and HI4320 were very similar to those reported by Kuan et al. (1). Interestingly, our strain presents two additional copies of *ucaA* and other fimbriae related-protein genes that are not present in HI4320, although they are present in other *P. mirabilis* strains. No plasmid sequences of HI4320 were found in Pr2921. The genomic sequence of this *P. mirabilis* strain along with that of other already-sequenced strains will be helpful to understand the role of fimbrial gene duplication and absence in the uropathogenic capacity of *P. mirabilis.*

### Nucleotide sequence accession numbers.

This whole-genome shotgun project has been deposited at DDBJ/EMBL/GenBank under the accession no. LGTA00000000. The version described in this paper is version LGTA01000000.
